# Supplementary material for: Comprehensive Investigation of Stereoselective Food Drug Interaction Potential of Resveratrol on Nine P450 and Six UGT Isoforms in Human Liver Microsomes
Source: Pharmaceutics. 2021 Sep 7;13(9):1419. doi: 10.3390/pharmaceutics13091419 (PMC8470274; doi:10.3390/pharmaceutics13091419)
Supplement: Supplementary file 1 [file pharmaceutics-13-01419-s001.zip › pharmaceutics-1337657-SM.pdf]

pharmaceutics

Supplementary Materials: Comprehensive Investigation of Stereoselective Food Drug Interaction Potential of Resveratrol on Nine P450 and Six UGT Isoforms in Human Liver Microsomes

Seung-Bae Ji+, So-Young Park+, Subin Bae+, Hyung-Ju Seo, Sin-Eun Kim, Gyung-Min Lee and Zhexue Wu,* Kwang-Hyeon Liu

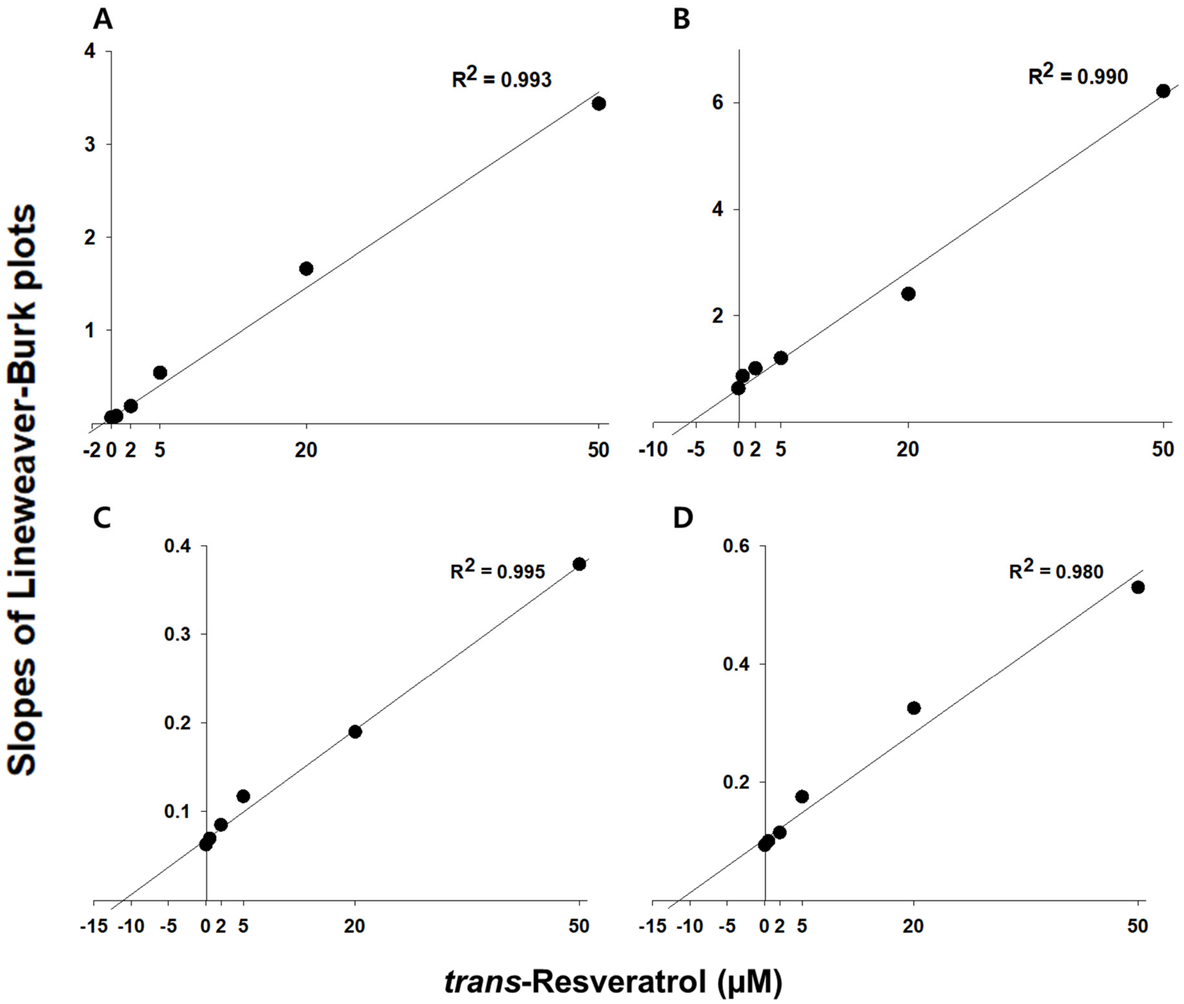

Figure S1. Representative secondary Lineweaver-Burk plots obtained from a kinetic study of CYP1A2-mediated phenacetin *O*-deethylation (**A**), CYP2C19-mediated *S*-mephenytoin hydroxylation (**B**), CYP2E1-mediated chlorzoxazone hydroxylation (**C**), and CYP3A-mediated nifedipine dehydrogenation (**D**) in the presence of different concentrations of *trans*-resveratrol (0, 0.5, 2.0, 5.0, 20, and 50 µM) in pooled human liver microsomes (HLMs).

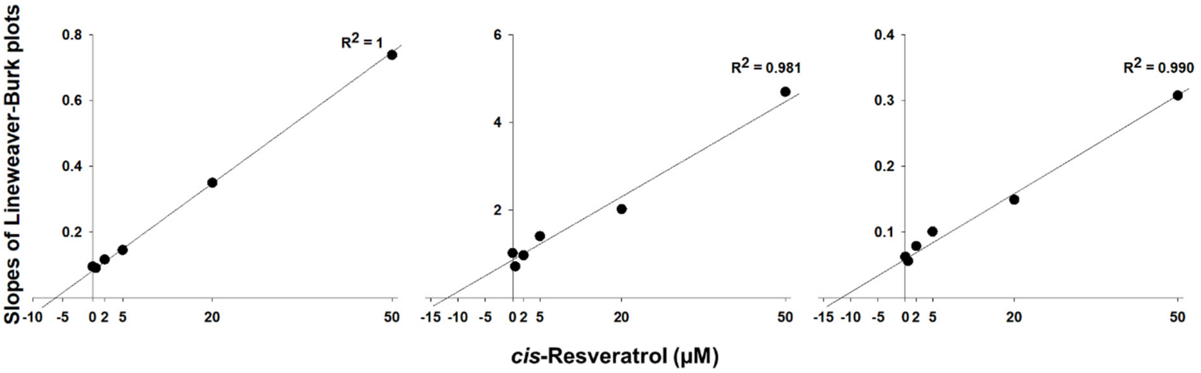

Figure S2. Representative secondary Lineweaver-Burk plots obtained from inhibition kinetic studies of CYP1A2-mediated phenacetin *O*-deethylation (**A**), CYP2C19-mediated *S*-mephenytoin hydroxylation (**B**), and CYP2E1-mediated chlorzoxazone hydroxylation (**C**) in the presence of different concentrations of *cis*-resveratrol (0, 0.5, 2.0, 5.0, 20, and 50 μM) in pooled human liver microsomes.
